# History of burns: The past, present and the future

**DOI:** 10.4103/2321-3868.143620

**Published:** 2014-10-25

**Authors:** Kwang Chear Lee, Kavita Joory, Naiem S. Moiemen

**Affiliations:** The Healing Foundation Burn Research Centre, University Hospital Birmingham Foundation Trust, Birmingham, UK

**Keywords:** Burns, history, future, fire disasters

## Abstract

Burn injuries are one of the most common and devastating afflictions on the human body. In this article we look back at how the treatment of burns has evolved over the centuries from a primarily topical therapy consisting of weird and wonderful topical concoctions in ancient times to one that spans multiple scientific fields of topical therapy, antibiotics, fluid resuscitation, skin excision and grafting, respiratory and metabolic care and nutrition. Most major advances in burn care occurred in the last 50 years, spurred on by wars and great fires. The use of systemic antibiotics and topical silver therapy greatly reduced sepsis related mortality. This along with the advent of antiseptic surgical techniques, burn depth classification and skin grafting allowed the excision and coverage of full-thickness burns which resulted in greatly improved survival rates. Advancements in the methods of assessing the surface area of burns paved way for more accurate fluid resuscitation, minimising the effects of shock and avoiding fluid over-loading. The introduction of metabolic care, nutritional support and care of inhalational injuries further improved the outcome of burn patients. We also briefly discuss some future directions in burn care such as the use of cell and pharmalogical therapies.

## Introduction

Burn injuries are amongst one of the most devastating of all injuries, having a great impact on the patients physically, physiologically and psychologically. Burns are still one of the top causes of death and disability in the world.[1] Physicians have searched for and formulated a myriad of treatments for burns over the centuries but these treatments mostly were of little benefit to the victims mainly because the fundamental understanding of the patho-physiological impact of burns was not known yet. There was an exponential increase in biomedical research and knowledge from the 18^th^ to early 20^th^ century in burn care, such as the recognition of the importance of burn surface area and skin grafting by Reverdin.[2] However, this was not reflected in improving survival and many patients still died of shock and infection. It was not until the past 50 years that the mortality of burns has been dramatically improved, thanks to the better understanding of the patho-physiology of burn injury.

**Table 1:** 

Access this article online	
**Quick Response Code**:	**Website**: www.burnstrauma.com
	**DOI**: 10.4103/2321-3868.143620

The treatment of burns is a major undertaking and involves many components from the initial first aid, assessment of the burn size and depth, fluid resuscitation, wound excision, grafting and coverage, infection control and nutritional support. Progress in each of these areas has contributed significantly to the overall enhanced survival of burn victims and this article aims to explore the history of burns to identify milestones and step-changes in each of these areas in the patient’s care. As in the case of the advancement in the treatment of trauma, these step-changes were mainly related to wars. Napoleon’s surgeon’s contributions to wound management that are still applicable today is an example. In burns, fire disasters as the Rialto fire in 1921 and Coconut Grove nightclubs fire in 1942 led to research that provided the first glimpse of the modern understanding of the patho-physiology of burns.

## Ancient burn treatments

The majority of ancient burn care consisted of topical therapies and can be traced back to centuries. One of the earliest records of burn treatment was described in an Egyptian Smith Papyrus written in 1600 BC which advocated the use of resin and honey salve for treating burns,[3] and the Ebers Papyrus in 1500 BC described the use of a wide variety of substances to treat burn wounds.[4] In 600 BC, Chinese described the use of tea leave abstracts and tinctures for burns.[3] Many famous philosophers and physicians have contributed to burn wound management such as Hippocrates, who in 400 BC, described the use of bulky dressings impregnated with rendered pig fat and resin with alternated warm vinegar soaks, augmented with tanning solutions made from oak bark[3] and in the 1^st^ century AD, Celsus described the use of wine and myrrh as a burn lotion, which had bacteriostatic properties.[3] The first description of first aid for burns was around 854 CE-925 CE, by Muhammad ibn Zakariya al-Razi (or otherwise known as Rhases in the west), an Arabian physician who recommended cold water for the relief of pain from burns.[5] A more recent example can be found in the 19^th^ century, where a mixture of linseed oil and lime-water (termed Carron oil) was being used to treat burns in Ironworkers in Scotland.[6]

With our current understanding of microbiology and infection, it would be difficult to comprehend why faeces and excrement, rich in disease causing pathogens, was used in the open wounds caused by burn. The use of a myriad of other different substances is interesting, however few of the ancient methods have any modern applications as they are unable to prevent bacterial infection which is one of the major causes of death of burn victims (the exception being vinegar, first described by Hippocrates but which is now being rediscovered for its antibacterial properties especially in the treatment of Pseudomonal infections).[7]

## Treatment of infection in burns

Burn injury damages the skin which is the primary barrier to infection. Damaged skin that provides a fertile ground to bacterial growth, together with immunosuppression that accompanies major burns, are the main contributors to wound infection, invasive sepsis and if not managed, death. With the advancement of resuscitation methods in burn patients, deaths due to hypovolemia and hyperosmolar shock are now uncommon. Meanwhile, sepsis is now the commonest cause of death following burn injury and contributes to almost 75–85% of all burn victim deaths.[8,9] Over the last few decades, many advancements have positively impacted on the incidence of burn wound infections and these include, topical and systemic antimicrobial therapies, early burn wound excision and closure and the introduction of infection control measures in modern burn units such as isolation facilities.

### Systemic antibiotics

The discovery of penicillin by the Scottish scientist, Sir Alexander Fleming in 1928 was a major breakthrough in the fight against microbial infections but it was not until World War II (WW2) that a way to manufacture the drug in large industrial scale was achieved. Penicillin played a crucial part in the treatment of the burn victims of the Coconut Grove fire in Boston in 1942, as it combated the Staphylococcus bacteria which typically led to toxic shock syndrome and also infected skin grafts. The emergence of methicillin-resistant staphylococcal strains however curbed the effectiveness of natural penicillins as the penicillinase produced by these bacteria hydrolyse the penicillin B-lactam ring and render them ineffective which in turn necessitated the development of penicillinase-resistant penicillins such as methicillin and cloxacillin. Streptococcal organisms are a major bane in the treatment of burn injuries as even the presence of a few B-hemolytic streptococci such as Group A (*Streptococcus pyogenes*) and Group B (*Streptococcus agalactiae*) can lead to a wound infection, loss of skin grafts and failure of a primary wound closure. Fortunately, natural penicillins remain effective and are bactericidal to these bacteria. Although staphyloccocal and streptococcal infections remain a major problem in burns, the landscape of microbes in burns continues to evolve with the emergence of antibiotic resistant strains *e.g.* the vancomycin-resistant enterococci (VRE) and *Pseudomonas spp.* which is now one of the most repeatedly encountered wound pathogen and a leading cause of noscomial infections in burn patients.

### Topical therapies

The aim of topical therapies has changed over the centuries as we understand increasingly more about the pathophysiology of burn wounds. In the early 20^th^ century, the goal of topical therapies was to prevent the release of ‘toxins’ from the burn wound and to dry out the wound to allow formation of a hard coagulum to minimize fluid loss. A variety of therapies were developed to achieve this such as the tannic acid spray described by Davidson in 1925[10] which was believed to produce a cleaner wound. However its use was stopped when it was found to be a hepatotoxic.[11]

One of the first topical antimicrobial treatments discovered was sodium hypochlorite (NaClO) in the 18^th^ century by Berthollet. Its use was hampered by irritation it caused,[12] but this was later discovered to be due to its variable quality and the free alkali or chlorine it contained. In 1915, Dr. Henry Dakin successfully developed a method of synthesizing hypochlorite without its irritating contaminants and found initially that a concentration of 0.5% was most effective as an antiseptic solution[13] (revised later to 0.025%[14]). This was further developed and used successfully in the treatment of burn wounds with a protocol of mechanical cleansing, surgical debridement and topical application of hypochlorite solution.

The major milestone in topical burn therapy was the application of solutions of silver compounds or salts, which played an important role in reducing the rate of burn wound sepsis and mortality. Silver sulfadiazine was developed by Charles Fox in the 1960’s[15] and has become the mainstay of topical antimicrobial therapy due to its success in controlling infection and minimal side effect profile. Mafenide acetate (Sulfamylon)[16] briefly was a viable alternative to the use of silver compound solutions in the treatment of infections but due to its carbonic anhydrase inhibitory effects which can lead to systemic acidosis, its use was all but discontinued except in cases of treatment of invasive wound infections. The other common silver-based therapy was silver nitrate, described by Moyer *et al.* in 1965.[17] Silver based topical treatments were successful in controlling infections especially *Pseudomonas aeroginosa* infections.

Recent development in dressing technology have seen the use of a variety of interesting materials incorporated into the dressing. There is emerging evidence for the use of dressings and gels[18–21] containing the naturally occurring glycosaminoglycan, chitin, which prevents early extension of burn injury[22], has antimicrobial properties,[23,24] promotes fibroblast proliferation and angiogenesis[25] and may promote burn wounds to heal, effects that are augmented by the incorporation of growth factors into the gel.[26,27] There has also been studies on the use of carbon fibre in dressings which has been shown to increase the absorptive capacity of the dressing, reduce inflammation, reduce bacterial growth and promote healing.[28,29]

### Role of non-pharmalogical therapies

Although antibiotic treatment is a major front in the war against infection, non-pharmalogical interventions play equally important roles, such as strict handwashing and hygenic nursing standards and patient isolation. The need for strict burn patient isolation became an important issue after WW2. State of the art burn centres were established in the United States then across the world. The Brooks Army center is an example of facility that was designed with infection control and patient isolation in mind.

## Surface area assessment in burns

It was only at the end of the 19^th^ century that it was realized that a relationship existed between the size of a burn and mortality. An early attempt at linking the size of burns to prognosis was carried out by Smart CB (1876) who studied 12 burn victims from an explosion aboard a ship and concluded that burn severity was determined by their size and depth, in addition to other bodily systems that were affected such as the airway.[30] Schjerning advanced this idea of the relation of mortality with burn size in 1884; he found that death always followed if two thirds of the body was burned, to be expected if 50% of the body was burned, and generally occurred if a third of the body was burned.[31]

However it was not until the late 18^th^ to early 19^th^ century was there any real attempt at accurately measuring burn size. Meeh in 1879 first described a method of using graph paper to measure burn body surface area (BSA).[32] Weidenfeld found this technique of measuring BSA too unwieldy and uncomfortable for the patients. By using his own and Meeh’s calculations, he discovered that there was a constant relationship of the surface area of well-defined body regions such as the head or arm and the total BSA. Using this knowledge he was then able much more accurately to indicate the extent of a burn injury and subsequently was able to correlate the size of a burn and the time of early death. Additionally he also found that other factors such as age, depth of burn and the general health (constitution) of the patient also played a role.[33]

Berkow calculated the surface area of various body parts in relation to the total body surface area, incorporating the use of calculations of body surface area as a function of overall height and weight by Du Bois D and Du Bois EF.[34] Berkow recognised as well that his formula needed to be corrected if used in children. Lund and Browder later modified this in 1944 by accurately defining the anatomical regions and dividing the BSA into 12 regions, as well as introducing an age correction factor for children based on a BSA calculations done by Boyd (1935) who in turn modified it from Du Bois D and Du Bois EF. The Lund and Browder chart is still widely used today. Notably, a Chinese scientist, Chu, estimated the bodily proportions in Chinese using a wet paper moulding method in 1982, however the differences between his findings and those of Lund and Browder were only slight, further supporting the accuracy of these two parallel methods.[35]

Berkow’s method not only gave rise to the Lund Browder chart but is also credited with a simplified method of assessing BSA which is the Rule of Nines. Wallace published details in 1951 of this simplified method to measure burn BSA.[36] This method is also sometimes credited to Pulaski and Tennison (especially in the USA), who had presented a very similar idea first in a symposium in 1950 and later combined his ideas with Wallace. The rule of nines estimates the patient’s hand surface area (defined as the area enclosed by a line drawn around the patient’s hand with extended fingers) as 1% of the BSA, although this has been shown as less than accurate.[37] However it has its place in the quick assessment of small burns.

The assessment of burn size is now a standard part of the diagnostic and treatment process in terms of fluid replacement therapy and plays a role as a prognostic indicator.

## Depth of burns classification

The first classification of burn depth actually goes back to the 16^th^ century. Guilhelmus Fabricus Hildanus, often regarded as the founder of surgery in Germany, in 1634 recognized the link between the length of time heats acts on the body and the resulting damage.[38] He categorised it into three stages; the first stage was characterised by erythema and blisters with colorless fluid, the second stage by erythema and blisters with yellow fluid, and the third, most serious stage, by the lack of blistering, and hard, dry skin that was blue or black and the lack of pain. Hildanus went on to publish De Combustionibus in 1670, which discussed the pathophysiology of burns and contributed to treatment of contractures. The three stage method of description was also utilized by Van Alberding in 1681, but he categorised it as light burns with blistering, contraction of the skin and thirdly, separation of the skin and underlying flesh with crust and ulcer formation.

The practice of classifying burns in ‘degrees’ was introduced in the 18^th^ century. Two German surgeons, Heister (1724) and Richter (1788) classified burns into four degrees:

- First degree: Heat, pain and small blisters.
- Second degree: Severe pain and large blisters.
- Third degree: Damage to the skin and underlying flesh, with crust formation.
- Fourth degree: Damage to all soft tissues down to the bone.

In 19^th^ century, Guillaume Dupuytren developed a classification of burn depth after a review of the care of 50 patients. This classification divided burns into the following six degrees:

- First degree: Erythema.
- Second degree: Skin inflammation with epidermal detachment.
- Third degree: Partial destruction of the papillary layer and subpapillary network of the corium.
- Fourth degree: Destruction of the skin down to the subcuticular layer.
- Fifth degree: Crust formation over skin and muscle.

Dupuytren’s classification is still in use by some today.[39] However many modern writings tend to use a simpler three degree classification system and this may be attributed to a French surgeon, Boyer, and was introduced in the beginning of the 18^th^ century (1814).[40] This classification divided burns into the following three degrees:

- First degree: Erythema.
- Second degree: Blistering of the skin leading to superficial ulceration.
- Third degree: Tissue disorganization leading to a dry yellow crust.

The current convention for describing burns is using depth rather than degrees; that is superficial, mixed depth and full thickness and new techniques are now in development to help physicians determine the depth of burns more accurately and objectively. These include thermal imaging[41], the use of laser techniques such as laser doppler imaging and laser speckle perfusion imaging[42] and also enhanced photographic methods which are aided by computer systems such as the spectrophotometric intracutaneous analysis scope.[43]

## Fluid resuscitation

The need for fluid resuscitation in burn injured patients was first recognised by Underhill. When he studied the composition of blister fluid of patients injured in the Rialto Theatre fire in 1921,[44] he found that it was similar to that of plasma and suggested that burn mortality may be due to loss of fluid rather than toxins. The introduction of methods of accurately quantifying burn surface area such as the Lund and Browder chart in 1944 and ‘rule of nines’ by Wallace and Tennison led to fluid replacement strategies based on total body surface area burned. By the 1940’s, the link between a proportional relationship between the percentage body surface area burned and the volume of fluid resuscitation required was established by Cope and Moore,[45] who were able to quantify the amount of fluid per area needed for adequate resuscitation by studying young adult burn victims in the Coconut Grove Nightclub in Boston in 1942. Moore went on to develop a formula for calculating the amount of replacement fluid required based on the percentage of body surface area burned.[46] Evans *et al.*, in 1952 added to this formula by including body weight in the calculations[47] and Reiss modified Evans formula by substituting normal saline with Ringer’s lactate and reducing the amount of colloid given.[48] Colloid was altogether removed by Baxter and Shires,[49] whose formula is now commonly known as the Parkland formula for fluid resuscitation which is still widely used in current practice.[49]

The use of colloidal solutions in the resuscitation of burns has had varied proponents and opposition over the years. Rosenquvist was the first to publish the use of dextran, a solution of polysaccharides in physiological saline, in the treatment of burn patients in 1947 and obtained good results with its use.[50] In 1962, Muir and Barclay proposed a formula of resuscitation which centered on the provision of colloid in the form of freeze dried plasma for resuscitation over a 36-hour period and this was used intensively in the United Kingdom (UK).[51] However Cochrane reviews in 1998 and 2003 (lastest version published in 2013), showed that there was no evidence from randomized controlled trials that resuscitation with colloids reduces the risk of death compared with crystalloids in patients with burns and discouraged their use.[52]

## Surgical interventions in burns

### Early excision of burns

The idea of excising burns existed in ancient times, in 1510–1590 AD, 400 years ahead of his time, Ambroise Pare, was one of the first to describe early burn wound excision. In 1607, Hildanus also recommended removal of incisions made into burn eschars to allow drainage of serous fluid and allow better penetration of medications. However the benefit of surgical eschar removal was hampered by poor hygiene and lack of antiseptic surgical techniques, which would lead to a high rate of infection and blood loss. Additionally, wound management was not fully understood. Therefore the practice of early burn excision was rightly abandoned even though physicians recognized the importance of the early removal of dead tissues to reduce the inflammatory response. Patients with full-thickness burns could only languish in hospitals while their eschars, which were invariably infected, sloughed off, leaving open wounds that heal by secondary intention with remarkable contractures and disabilities.

Significant advances in infection control made burn eschar excision possible, when Lister in 1865 started successfully utilising carbolic acid (or phenol) as a method to sterilise surgical instruments and clean wounds. This, along with advancements in topical infection control, led to a significant decrease in post-operative infections and improved survival. On the other hand, the understanding and management of burn shock in the late 1950s was already underway. Improvements in the treatment of shock such as improved fluid resuscitation techniques, blood volume monitoring and realisation of the importance of urine output measurement allowed a greater efficiency of burn shock treatment and consequently more patients survived the initial shock stage. This in turn allowed the excision of full thickness burns to be more feasible and safe.

WW2 brought about a tremendous increase in burn victims, and physicians had to find a way to help patients recover faster. Physicians approached the problem of full thickness burns initially by chemical debridement with pyruvic acid and starch, which allowed grafting as early as 6 days post debridement.[53] Several other authors reported in the 1940s their successful attempts at surgical excision of full thickness burns.[54–57] However most reports have been anecdotal.

In 1960, Jackson *et al.*, published a series of pilot and controlled trials which showed that excision and grafting (20–30% of TBSA) could be safely achieved as long as shock was controlled via red cell volume monitoring[58] and recommended the technique in cases of full-thickness burns (as natural slough separation would take 6 weeks), burns of 15–20% and deep circumferential burns of the trunk which affect respiration. However due to small numbers, they could not conclusively demonstrate an impact of the technique on mortality, infection nor healing time which probably prevented it from being accepted by the majority of surgeons at the time. What probably changed practice was the introduction of the tangential excision technique by Zora Janzekovic in the 1970s which was a significant improvement in the technique.[59] An important modification introduced by this technique was that the excision not only included slough but also of the damaged dermis down to bleeding tissues. Her technique was advocated by many, several (including herself)[59] who have reported improvement in mortality and also decrease in hospital stay when compared with conservative treatment.[60,61] The technique became the standard of care in most leading burn centres across the globe, but was only limited to small burns that could be covered by skin from patients’ own donor sites.

Wound cover after major burn excision was the challenge that delayed the practice till the 1970’s, when xenograft, pig skin, and cadaver skin became more widely available. Although Bettman reported success in the treatment of children with large full-thickness burn injuries with allograft skin in way back in 1938[62], modern day skin bank only began following the establishment of the United States Navy tissue bank in 1949.[63]

In the first publication of early burn excision of major burns, Tompkins *et al.*, credited the introduction of early excision and grafting of large burns for the dramatic decrease in mortality in children from 24% to 27%.[64] Dr. David Herndon in a series of landmark papers has demonstrated the benefit of total early burn wound excisions for survival and improved outcomes.[61,64]

### Skin grafting and the development of skin substitutes

The earliest record of skin grafting goes back to the 5^th^ century AD, where an Indian surgeon, Sushrutha, repaired noses, that were amputated as punishment for crimes, using strips of skin from the forehead which were flapped downwards and grafted over the wound[65,66]. Sushrutha has also been documented to transplant skin from the buttock to the nose. The first documentation of a modern skin graft in humans was by Carl Bunger in 1823. This again involved a nose wound, and full thickness skin from the inner thigh was used for this purpose. During this time however, the success of skin grafts was low due to inefficient harvesting and use of large and thick grafts. Free skin grafting was successfully reproduced by Reverdin, who was still a student at the time, in 1869 to encourage healing and closure of slow healing or chronic wounds.[2] Reverdin utilized “pinch grafts”, which were small circular skin discs obtained by pinching a small amount of skin and cutting it out. This was done repeatedly to produce islands of small grafts that were used to cover the wound, and it left donor sites that healed quickly due to their small sizes. This was soon popularized in England by George Pollock in 1870.[67] Advances in the quality of surgical instruments meant that thinner grafts than previously possible could be harvested. Thiersch took advantage of this and developed and advocated the use of razor thin skin flaps or “razor flaps”[68] in 1874. However these grafts did not produce satisfactory results in general and were limited to the treatment of small ulcerated wounds. Seven years later, Girdner reported the first successful use of allogeneic skin in burn wound coverage.[69]

In the 1920’s, Blair and Brown discovered that deep islands of hair follicles and sebaceous gland epithelial cells were the factor responsible for initiating the healing at donor sites. This meant that grafts could be harvested to different depths as long as these islands were preserved. Tools that allowed the surgeon to control the depth of skin harvested then quickly developed. Surgeons initially had to harvest thin grafts with the use of blades that afforded no mechanism to control graft thickness such as the Blair and Catlin knives.[70] Hofman and Finochietto developed knives that permitted precise regulation of the thickness harvested via screw-adjusted knives.[71] Split thickness skin grafts (so called as the tools used to harvest these grafts resembled the tools used for splitting leather) however only started becoming more popular in the 1930s due in part to the development and availability of more reliable instruments. Humby then developed a knife allowed control over the depth harvested[71] in 1936, followed by an adjustable dermatome by E.C Padgett in 1939.

The method of meshing grafts can be traced back to Lanz in 1907, who designed a cutting tool consisting of a series of small knives mounted in parallel to make multiple holes in a graft, forming a mesh.[72] A method of expanding the graft size was described by Meek in 1958[73] which utilizes a special dermatome (Meek Wall dermatome) and prefolded gauzes which allowed a nine fold expansion of the harvested graft.[74] The Meek dermatome was however viewed as cumbersome and required much skill and was thus superseded in 1964 by the introduction of simpler ‘mesh dermatome’ developed by J.C. Tanner *et al.* which allowed a graft to be expanded three times the original donor site size.[75] The Meek technique has in recent years though been seeing a revival in its use including the development of modified, air driven dermatome by Kreis *et al.*, in 1994 and has an advantage over mesh grafts when donor sites are limited *e.g.* in larger burn wounds.[76–78] Additionally, a study investigating the real expansion rate of meshers and Meek micrografts showed the Meek technique provided more reliable and valid expansion rates compared to the skin meshers.[74,79]

Despite the introduction of meshing and micrografting techniques, these were still insufficient to meet the increased demand for skin in cases of large burns especially with the introduction of the concept of early total burn excision, and thus cryopreservation and long term storage of human skin for both autologous and allogenic skin transplantation were developed in the 1940–50’s[80,81], of which a major milestone was the introduction of the use of glycerol to cryopreserve skin.[82]

Skin allografts allowed the coverage of burns in cases where there was extensive skin loss or where limited sources of sites for autograft harvesting were available and could be used with or without concurrent skin autografts. Jackson described a combined grafting technique[83] which utilized alternate placement of narrow stripes of allograft and autograft onto a granulating or excised wound hence it was also known as ‘Tiger-striping’. Following the adherence of the grafts, a process termed ‘creeping substitution’ occurs, whereby migration of autologous epithelial cells occurs across the wound surface between the sheets of autograft and underneath the allograft sheet causes the allograft sheet to slowly lift and separate, leaving an epithelized wound bed. This technique has been used and adapted by the Chinese surgeons, Zhang and co-workers,[84–86] who minced autologous skin into pieces less than 1 mm in diameter and then seed these ‘micrografts’, into the dermis of large sheets of allograft skin prior to applying it onto the wound. The autograft epidermal cells proliferate and migrate and eventually via the process of creeping substitution, lifts and separates the allograft. While this method results in effective skin expansion ratios up to 1:18, it has been associated with severe wound contraction compared to sheet autografts.[87] Currently, micrografting techniques including the Meek technique has been more widely adopted in China than any other country.

Unfortunately, allogenic skin transplants trigger a potent reaction and rejection by the host immune skin and are invariably rejected acutely and immunosuppressive treatments that have proven effective in preventing rejection of organ transplants have sadly little or no effect on skin transplants. Thus in the UK, the use of allografts has been slowly phased out.

Another method of wound cover had to be developed and in the 1970’s, Yannas, a scientist at the Massachusetts Institute of Technology and Burke, a surgeon at the Massachusetts General Hospital and Shriners Burns center collaborated to develop and produce the first artificial skin, Integra®. It consisted of two layers, a collagen-chondrotin matrix and a silicon layer on top. The first multicentered clinical trial was conducted and published in 1988[88] and was granted an FDA licence in 1996. Integra is now widely used in acute burns and reconstructive surgery. Other artificial skin substitutes include Apligraf, developed by Bell *et al.*, and Matriderm by Otto Suwelack.[89]

## Metabolic care and nutritional support

The catabolic response to injury has been noted as early as in the 18^th^ century by Sir John Hunter, but the ‘ebb’ and ‘flow’ phases of injury was first clearly documented by a Scottish nutritional scientist, David Cuthbertson in the early 1940s while he was studying the urinary excretion of minerals in facture patients.[90] The ‘ebb’ phase in burns (also known as the acute or shock phase) occurs within 48 h of injury and is characterized by a decrease in the metabolic rate with depressed oxygen consumption, cardiac output and glucose tolerance.[91] If patients survive this shock phase, they then go into the ‘flow’ or chronic response phase which is typically characterized by an elevated hyperdynamic state with increased metabolism and cardiac output.[91] This increased hypermetabolism is responsible for the highly catabolism seen in severe burn injuries with accelerated glycolysis, proteolysis, and lipolysis leading to weight loss and the erosion of lean body mass (LBM), generalized fatigue and a weakened immune response.[92,93] The loss of LBM has a particularly devastating effect on the outcome of patients as Chang *et al.*, has shown that impaired immunity is seen with 10% loss in LBM, decreased wound healing with 20% loss in LBM and death with 40% loss of LBM.[94]

Several therapeutic strategies have been devised to ameliorate the hypermetabolic response including environmental control, excision of injured and dead tissue to reduce inflammatory stimulation, nutritional support and pharmacological therapies.

Only since the early 1900s has it been recognized that burn patients require an increased caloric intake. High caloric feeding was advocated by Shaffer *et al.* in 1909[95] and more recently by Wilmore in 1979.[96] An in-depth understanding of the importance of the nutrition support was only established in 1970s. A high protein and high calorie diet supplemented with multivitamins and trace elements are required for burn patients. The evidence of other dietary supplements such as glutamine is not firmly established yet.[97–99] The route of feeding has been shown to be important as well. Herndon *et al.* showed that nutritional support, when given parentally, was shown to be harmful as it increased both immune deficiency and mortality and thus continuous enteral feeding is advocated instead.[100] A recent review of the evidence surrounding nutritional support for burns patients guided the recommendations made by the European Society for Clinical Nutrition and Metabolism (ESPEN), including the need for early enteral feeding via the nasojejunal or nasoduodenal route.[101]

In the last few decades, various pharmalogical interventions have been trialled in burn patients, chiefly to attempt to reduce the hypermetabolic response. Examples include Propranolol (a B-receptor antagonist),[102,103] anabolic agents such as human recombinant growth hormone[104] and oxandrolone,[105] and anti-hyperglycaemic agents such as insulin[106,107] and metformin.[108]

The loss of LBM can also be accomplished by early rehabilitation from day one after the injury with active rather than passive exercises, even with intubated patients, will preserve the LBM and reduce complications.

## Inhalation injury in burns

The interest in pulmonary function in burns patients probably started in the 1970s when physicans started to note that pulmonary complications were common in burn patients. With improvements in the treatment of burn shock and sepsis, inhalational injury has now replaced these two causes as the main cause of mortality in burn patients.[109] Inhalational injury by itself has been shown to be associated with pulmonary dysfunction for at least 6 months after the injury.[109]

Pulmonary complications in burn patients can arise from direct injury to the respiratory tract via the inhalation of heated air and chemicals released by combustion, and also iatrogenic factors such as fluid-overloading during resuscitation and lung damage by mechanical ventilation.

Airway and pulmonary inflammation can also result from smoke inhalation alone. An autopsy study by Zikria *et al.*, in 1972 showed that 70% of all burn victims who died within 12 hours of injury had inhalational injury which could be linked to the toxic products of combustion.[110] A study by Herndon *et al.*, in 1985 using an experimental sheep model of smoke inhalation injury showed that the pulmonary edema that occurred after smoke inhalation was the result of an increase in microvascular permeability and hypothesized that this may be secondary to neutrophil degradation.[111] The global immunosuppression that accompanies burn injuries increases the risk of developing respiratory tract infections.[112]

The treatment of burns itself can contribute to the development of lung injury. Moore *et al.*, noted that although fluid resuscitation and blood transfusions prevented acute renal failure in trauma patients, these patients went on to develop pulmonary complications.[113] Pruitt *et al.*, hypothesized that pulmonary insufficiency in burns patients was due to a complex mechanism of interstitial edema leading to alveolar epithelial cell (type 2) damage and pulmonary circulation constriction secondary to vasoactive substances.[114] Achauer *et al.*, proposed a number of measures to prevent pulmonary edema including the use of pulmonary artery wedge and central venous pressure monitoring and also the supplementation of crystalloids with albumin to reduce the amount of fluid required.[115] This was supported by animal studies by Holleman *et al.*, which found that animals that were given only crystalloids post-scald injury had a higher water content in their lungs and recommended the addition of colloids to resuscitation fluid.[116] The view on using colloids however, has changed in the last 40 years. Moncrief, from the U.S. Army of Surgical Research, hypothesized that the use of colloid was of no benefit in the first 24 hours due to the disturbed capillary permeability.[117]

In addition to overhydration, Moore *et al.*,[113] also recognized that inhalational lung injury could be exacerbated by tracheostomy and mechanical ventilation at high oxygen tension. Traditionally, mechanical ventilation is acheived using tidal volumes of 10–15 ml per kilogram of body weight which is larger than in normal subjects at rest (7–8 ml per kilogram)[118], which can lead to an excessive distension of the lung leading to disruption of the pulmonary epithelium and endothelium, and the release of inflammatory mediators.[119,120] The use of lower tidal volumes (TV) during ventilation of patients with acute lung injury and acute respiratory distress syndrome has been shown by a landmark study by the Acute Respiratory Distress Syndrome Network in 2000 to reduce mortality by 22% and increase the number of ventilator-free days. There is evidence as well that low TV ventilation protects against pulmonary complications in patients without acute respiratory distress syndrome.[121] Most burn centers now adopt this low TV approach to reduce ventilator-induced injury.[122]

## Future

The last 50 years has seen a tremendous improvement in the advancement of burn treatment with a significant reduction in mortality which can be attributed to the developments in early burn excision, early fluid resuscitation, infection control and nutrition. There is still many areas in burn care left to explore and improve and here we highlight some of the interesting developments in the field of burn care.

### Wound cover with cell therapy

Human keratinocytes can be cultured and are currently being used as an adjunct to burn wound healing.[123,124] Stem cells have been shown to accelerate burn wound healing in animal models[125] may prove to revolutionize burn care. Early studies with ‘bioprinted’ scaffold incorporating bone marrow-derived mesenchyme derived stem cells and amniotic fluid-derived stem cells showed promising results in a wound healing model but these were attributed to the growth factors secreted by the stem cells which did not become incorporated into the healing tissue.[126] This concept however may perhaps help to allay fears regarding the use of pluripotent cells and the potential for malignant transformation. Another exciting development in the field of stem cells in wound healing is the concept of healing without scarring as has been demonstrated in early fetal life.

Research is currently underway to unlock the molecular mechanisms underlying this process with the hope that this can be translated into clinical practice.[127]

### Pharmalogical and nutritional interventions

Recent advancement in pharmacological interventions include the use of statins, which have demonstrated anti-inflammatory properties alongside their ability to lower serum lipid concentrations. They have been shown to improve survival in an animal model of burns sepsis[128] and there is some evidence that this benefit translates to patients.[129–131]

Systemic administration of erythropoietin (EPO) has been shown to decrease secondary evolution of experimental burn injuries[132] and may therefore have a role in minor burns. However it has to be noted that recombinant EPO has been shown to be ineffective in preventing the anemia that occurs after a major burn injury and does not reduce the need for ongoing blood transfusions.[133] This resistance to EPO treatment may be due to dampened erythropoiesis and lymphopoiesis and enhanced myelopoiesis in the bone marrow as demonstrated following burn injury in animal models.[134]

### Shock wave therapy

There have been promising early clinical results on the use of shock-wave therapy (ESWT) for healing burn wounds.[135,136] Animal studies have demonstrated dampening of the inflammatory response with a single dose of ESWL post-burn[137] and expedition of healing with multiple doses[138] as well as improved angiogenesis and blood flow along with increased numbers of rolling and sticking leukocytes.[139] A large phase III clinical trial to further evaluate the use of this treatment modality in burns patients is currently underway.

## Conclusion

In this article we have highlighted the major advances in the evolution of the care of burns patients over the past 3500 years. The advancement of burn care has been closely associated with our deeper understanding of its pathophysiology; we have now come to understand the impact that burn injuries have in the multiple fields of current medical science i.e. in metabolism and circulation, electrolyte balance and nutrition, immunology and infection, inflammation, pulmonary function and wound healing. The major advancement in burn care in this century, especially in the last 50 years, is a typical example of the positive feed-back between in-depth research and the improvement of clinical care. It has transformed from the limited attempts to prevent complications in the first half of the century to the current state of effective shock therapy and surgical interventions which has led to a massive increase in the survival of burn victims. Despite this, many challenges still remain and the focus of burns care in the future will be to overcome the problem of burns in the elderly, extensive burns, to improve the quality of the lives saved by shortening healing times and therefore lengths of hospital stay and to improve scarring. It is hoped that new technologies and advances in wound care will achieve wound cover with minimal scarring.
